# Molecular characterization of a novel putative pathogen, *Streptococcus nakanoensis* sp. nov., isolated from sputum culture

**DOI:** 10.1128/spectrum.01354-24

**Published:** 2024-09-13

**Authors:** Takeaki Wajima, Takashi Sugawara, Emi Tanaka, Kei-ichi Uchiya

**Affiliations:** 1 Department of Microbiology, Faculty of Pharmacy, Meijo University, Nagoya, Japan; 2 Tokyo General Hospital, Tokyo, Japan; The Ohio State University College of Dentistry, Columbus, Ohio, USA

**Keywords:** *Streptococcus*, multidrug resistance, pathogenesis

## Abstract

Reports of novel species of α-hemolytic *Streptococcus* have increased recently. However, limited information exists regarding the pathogenicity of these species, with the exception of *Streptococcus pneumoniae* and *Streptococcus pseudopneumoniae*. In this study, a quinolone-resistant α-*Streptococcus* strain, MTG105, was isolated from the sputum of a patient with pneumonia. This strain was first identified as *S. pneumoniae* at the hospital laboratory; however, it exhibited unique genetic features upon further analysis. Digital DNA-DNA hybridization and average nucleotide identity based on BLAST values from whole-genome sequencing revealed MTG105 to be a novel species closely related to *S. pseudopneumoniae*. Although MTG105 carried two copies of the pneumolysin gene, similar to *S. pseudopneumoniae*, this isolate exhibited susceptibility to optochin under both aerobic and 5% CO_2_ conditions. Notably, no biochemical features could be used to definitively identify this species. In an infection assay using organotypic lung tissue models, MTG105 induced epithelial damage comparable to that of *S. pneumoniae* and *S. pseudopneumoniae*, possibly suggesting its potential as a pathogenic α-*Streptococcus*. The natural transformation abilities of *Streptococcus* species facilitate their exchange of genes within the same genus, resulting in the existence of species with increasingly more diverse genome structures. Therefore, the identification of this species highlights the importance of monitoring the emergence of novel species exhibiting virulence and/or multidrug resistance. This isolate was proposed as a novel species, designated *Streptococcus nakanoensis* sp. nov. The type strain was MTG 105^T^ (= JCM 35953^T^ = CCUG 76894^T^).

**IMPORTANCE:**

The genus *Streptococcus* encompasses a wide range of bacteria with more than 60 species. Recently, there has been a notable increase in reports of novel species of α-*Streptococcus* based on genomic analysis data. However, limited information exists regarding the pathogenicity of these species. In this study, a quinolone-resistant α-hemolytic *Streptococcus* strain, MTG105, was isolated from a patient with pneumonia. Genetic analysis revealed that this species was a novel species closely related to *S. pseudopneumoniae*. In an infection assay using organotypic lung tissue models, MTG105 induced epithelial damage comparable to that caused by *S. pneumoniae* and *S. pseudopneumoniae*, strongly suggesting its potential as a pathogenic α-*Streptococcus*. The natural transformation abilities of *Streptococcus* species facilitate gene exchange within the same genus, leading to the emergence of species with increasingly diverse genome structures. Therefore, the identification of this species underscores the importance of monitoring the emergence of novel species exhibiting virulence and/or multidrug resistance.

## INTRODUCTION

The genus *Streptococcus* encompasses both commensal and pathogenic bacteria found in humans and animals, with more than 60 species identified (1–3). These species have been categorized based on Lancefield grouping, hemolytic pattern, and DNA sequence (2, 4, 5). *Streptococcus* are classified into α, β, and γ types based on hemolytic patterns on blood agar. In addition, *Streptococcus* are categorized into six major clusters—mitis, anginosus, slivarius, bovis, mutans, and pyogenic groups—based on phylogenetic analysis using 16S rRNA sequence (2, 6). Among these clusters, the mitis group is prominent among α-*Streptococcus* isolated from humans. *Streptococcus pneumoniae* and *Streptococcus pseudopneumoniae* are well-characterized human pathogens, whereas other Streptococci, such as *Streptococcus mitis* and *Streptococcus oralis*, typically function as commensal bacteria, forming part of the human normal flora, such as the oral cavity flora. Recently, reports of novel species of α-*Streptococcus* based on genomic analysis data have seen a notable upsurge. Over the past few years, more than 10 new species isolated from humans have been proposed (7–23). Many of these novel α-*Streptococcus* species share similarities with *S. mitis*. However, few species of pathogenic α-*Streptococcus,* such as *S. pneumoniae* and *S. pseudopneumoniae,* have been identified.

Animal experiments are a predominant method employed in pathogenic evaluation. However, they present difficulties when assessing infection response for human pathogens owing to host specificity and ethical concerns. To address these issues, three-dimensional (3D) human organotypic tissue models and organoid technology have been developed (24–26). These models circumvent issues related to host specificity and ethical concerns. Moreover, several studies have demonstrated that these models can effectively replicate human responses and are valuable tools for evaluating bacterial pathogenicity (24, 25, 27).

In this study, a quinolone-resistant α-hemolytic *Streptococcus* strain, MTG105, was isolated from the sputum sample of a patient with pneumonia, and its genetic features and pathogenicity were characterized using whole-genome analysis and 3D organotypic lung models, respectively.

## MATERIALS AND METHODS

### Bacterial isolate

The α-hemolytic *Streptococcus* strain MTG105 was isolated from the sputum sample (Miller and Jones' classification, P1; Geckler quality of 3) of a male patient in his 90s with a respiratory infection, collected at the clinical laboratory of Tokyo General Hospital in 2021. This isolate was cultured on trypticase soy agar with 5% sheep blood and identified as quinolone-resistant *S. pneumoniae* using MicroScan AutoSCAN-4 (Beckman Coulter, Brea, CA, USA) in the clinical laboratory. Subsequently, without disclosing the patient’s personal information, the isolate was transferred to Meijo University for a detailed investigation into the mechanism of quinolone resistance.

### Genomic analysis

The genomic DNA of MTG105 was extracted using the Wizard Genomic DNA Purification Kit (Promega, Madison, WI, USA). Genome sequencing was conducted using both GridION (Oxford Nanopore Technologies, Oxford, UK) and DNB Seq-G400 (MGI Tech, Shenzhen, China) according to the manufacturer’s instructions at the Bioengineering Lab in Kanagawa, Japan. The resulting sequence data were assembled using Unicycler ver. 0.4.7 (https://github.com/rrwick/Unicycler), with default parameters. The quality of the genome sequences was assessed using CheckM ver. 1.0.12 (https://github.com/Ecogenomics/CheckM). Subsequently, annotation and analysis of the genome sequences were conducted using DFAST version 1.2.18 https://dfast.ddbj.nig.ac.jp/), with default parameters. Digital DNA-DNA hybridization (dDDH) and average nucleotide identity based on BLAST (ANIb) values were calculated using the Type Strain Genome Server (TYGS) website (https://tygs.dsmz.de/) and JSpeciesWS (https://jspecies.ribohost.com/jspeciesws/), respectively.

The genome project and samples are indexed at the DNA Data Bank of Japan (DDBJ) under BioProject PRJDB14936 and BioSample SAMD00566859. The obtained and annotated sequence data have been deposited in the DDBJ under DDBJ/EMBL/GenBank accession numbers AP027139 (whole genome) and LC744565 (*16S rRNA* sequence).

### Antimicrobial susceptibility

Antimicrobial susceptibility was assessed using a broth dilution test, according to the guidelines of the Clinical and Laboratory Standards Institute. *S. pneumoniae* ATCC 49619 and *S. mitis* JCM 12971^T^ were used as quality controls. The antimicrobial agents used for susceptibility testing included benzylpenicillin (FUJIFILM-Wako Chemical, Osaka, Japan), ampicillin (FUJIFILM-Wako Chemical, Osaka, Japan), cefotaxime (FUJIFILM-Wako Chemical), meropenem (Tokyo Chemical Industry, Tokyo, Japan), tetracycline (FUJIFILM-Wako Chemical), levofloxacin (Tokyo Chemical Industry), ciprofloxacin (Tokyo Chemical Industry), clarithromycin (Tokyo Chemical Industry), azithromycin (Tokyo Chemical Industry), vancomycin (FUJIFILM-Wako Chemical), and rifampicin (FUJIFILM-Wako Chemical).

### Biochemical characterization

Biochemical characteristics were assessed using Rapid ID 32 Strep (bioMérieux, l'Etoile, France) and API ZYM (bioMérieux). The preparation and determination methods were based on the protocols outlined in the manufacturer’s instructions. Susceptibility to optochin was evaluated using optochin discs (Eiken Chemical, Tokyo, Japan) under both ambient air and 5% CO_2_ conditions. The bile solubility test was performed as previously described (28).

### Mass spectrometry

The sample was prepared by ethanol/formic acid extraction (21). The spectrum was recorded with a Matrix-assisted laser desorption/ionization-time of flight (MALDI-TOF)-MS Bruker autoflex maX mass spectrometer and analyzed using MALDI Biotyper Compass Explorer ver. 4.1.60 (Bruker, Billerica, MA, USA).

### Phylogenetic analysis

For phylogenetic analysis, the 16S rDNA nucleotide sequence was extracted from the whole-genome data of strain MTG105. Additional sequences for alignment and homology estimation were retrieved from the DDBJ database. Multiple-sequence alignments of the retrieved DNA sequences, calculation of phylogenetic distance, and construction of a phylogenetic tree were performed using Mega-X software (https://www.megasoftware.net/).

### Infection assay using 3D organotypic lung tissue models

A 3D organotypic lung model was constructed following previously described protocols (24). Briefly, the collagen matrix (Nippi, Tokyo, Japan) was solidified in a six-well culture insert (Corning, NY, USA). Subsequently, the lung fibroblast cell line, MRC-5 (Riken BRC, Tsukuba, Japan), was incorporated into the collagen matrix and layered. After solidification, minimal essential medium α (FUJIFILM-Wako Chemical) supplemented with 10% fetal bovine serum was added to both the top and bottom compartments of the insert. After incubation at 37°C for 7 days, the human bronchial epithelial cell line 16HBE14o- (Merck, Darmstadt, Germany) was seeded onto the top surface of the model and cultured on the medium for an additional 3 days. The resulting model was exposed to air for 7 days. For infection studies, the constructed model was inoculated with 50 µL of cultured bacteria and incubated at 37°C for 24 h.

## RESULTS

### Quinolone-resistant α-hemolytic *Streptococcus* clinical isolate, MTG105

The α-hemolytic *Streptococcus* species MTG105 was isolated from the sputum sample of an inpatient with pneumonia. Routine species identification tests performed using MicroScan AutoSCAN-4 at the clinical laboratory of Tokyo General Hospital classified this isolate as *S. pneumoniae*. At the same time, a smaller number of other α-Streptococci were also isolated but they were considered commensal bacteria. MTG105 exhibited resistance to tetracycline, levofloxacin, ciprofloxacin, and clarithromycin (Table S1). Given the comparative rarity of quinolone-resistant *S. pneumoniae* in Japan, this isolate was subjected to further analysis in the laboratory of Meijo University. To analyze quinolone resistance, *gyrA* and *parC* were amplified using primers specific to *S. pneumoniae* (29). However, no amplicons were detected, suggesting that this isolate possessed unique genetic features.

### Genomic analysis

**FIG 1:**
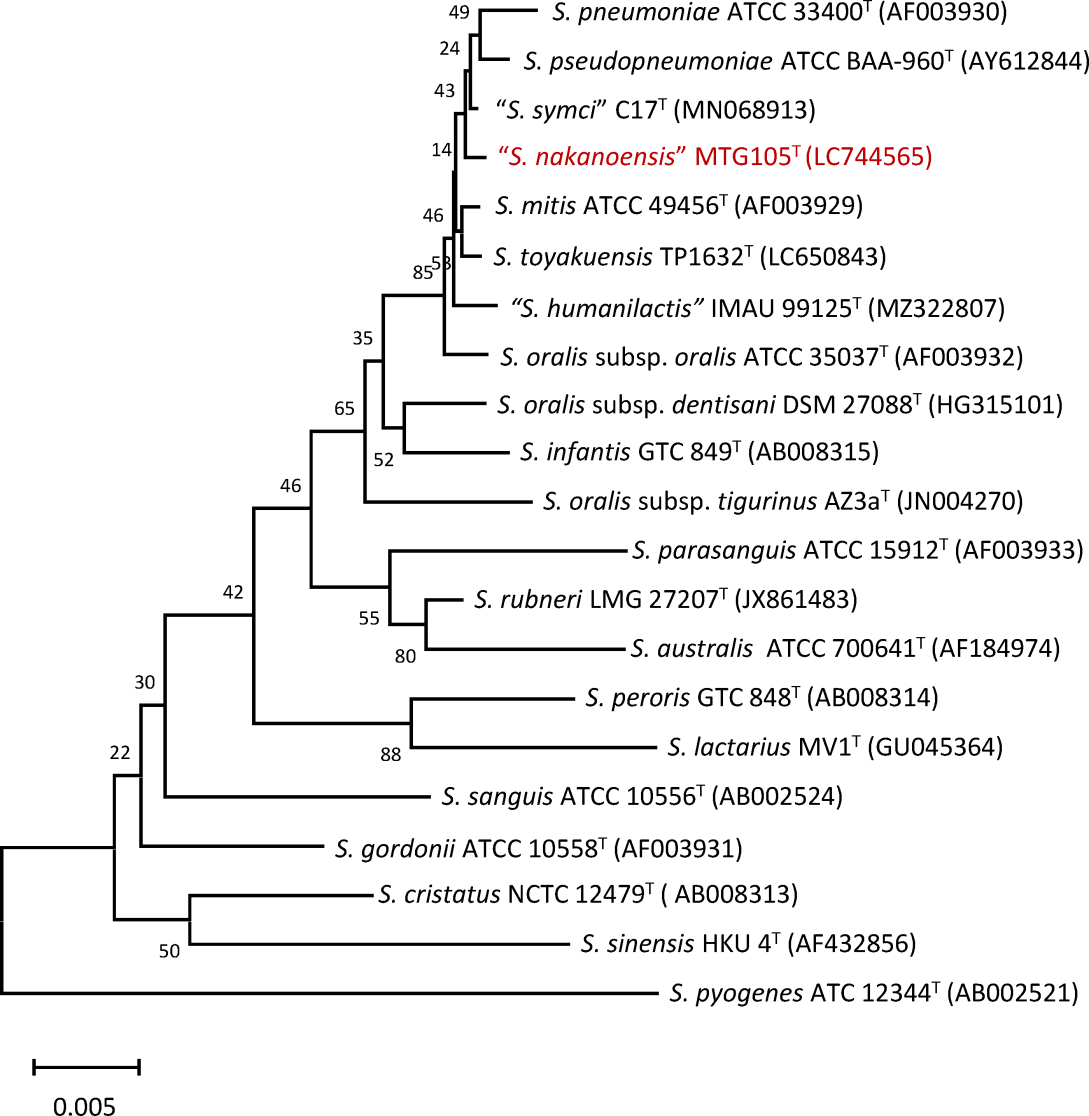
Phylogenetic analysis of *Streptococcus* species. A neighbor-joining dendrogram based on Tamura–Nei’s genetic distance of 16S rDNA sequences was constructed using MEGA-X software.

**FIG 2:**
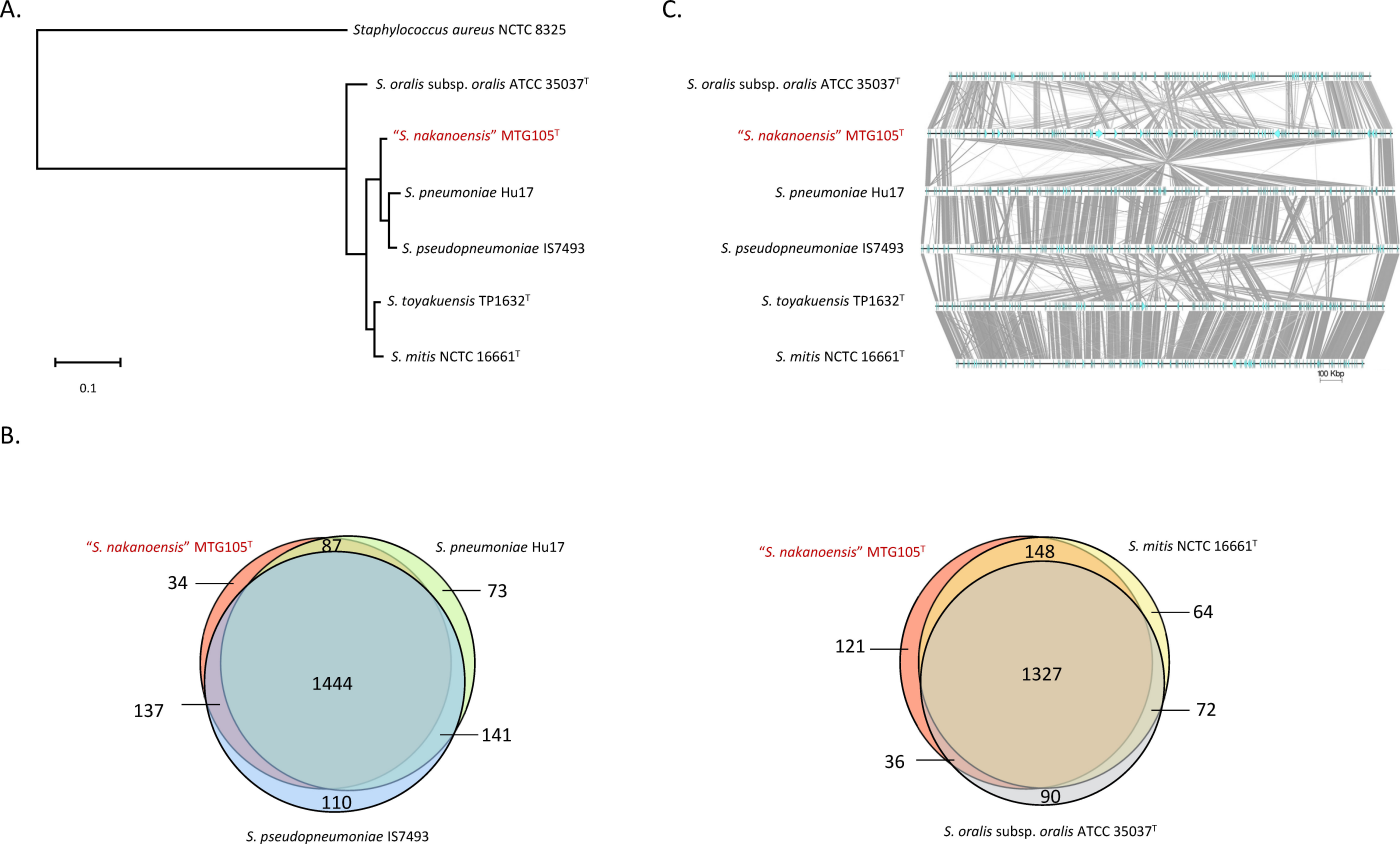
Whole-genome comparison analyses. (A) Dendrogram based on whole-genome ortholog using OrthoFinder algorithm. (B) Venn diagram of ortholog using OrthoVenn2 algorithm. (C) Comparative genomic analysis using EasyFig program. Single strain per species was included. In case genome sequence was available, type strains were included. Otherwise, the strain was randomly selected from available genome sequences.

To elucidate the genetic features of MTG105, whole-genome sequencing was performed using both DNB Seq-G400 and GridION. Sequencing achieved a genome coverage of 1,983× and hybrid assembly yielded a complete closed genome sequence, revealing a closed genome size of 2,127,737 bp. The genome size comprised 1,895 coding sequences, 12 rRNA, and 59 tRNA, with a guanine-cytosine (GC) content of 40.4%. Comparative analysis of *gyrA* and *parC* sequences with those of *S. pneumoniae* revealed homologies of 93.60% and 91.42%, respectively (Fig. S1). Species identification based on the whole-genome sequence was subsequently performed. The dDDH value, calculated using the TYGS server, was highest at 56.0% (for *S. pseudopneumoniae* ATCC BAA-960^T^) (Table 1). In addition, the highest ANIb value, calculated by JSpeciesWS, was 93.69 (for *S. pseudopneumoniae* ATCC BAA-960^T^) (Table 1). Given that the dDDH and ANIb values were less than 70% and 95%, respectively, this isolate was suggested to be a novel species. Phylogenetic analysis using 16S rRNA sequences indicated that this isolate belonged to the mitis group and was closely related to *S. pneumoniae* and *S. pseudopneumoniae* (Fig. 1). For a detailed characterization of the genetic features of MTG105, comparative genomic analyses were performed using related species belonging to the mitis group (Fig. 2). MTG105 was classified into a close cluster of *S. pneumoniae* and *S. pseudopneumoniae* (Fig. 2A) but exhibited 34 unique orthologs compared with these species (Fig. 2B). The genome of MTG105 exhibited considerable divergence compared with that of *S. pneumoniae* (Fig. 2C).

**TABLE 1:** Comparison of the genetic distance based on whole-genome sequence^*a*^

Query strain	Subject strain	dDDH		G + C content difference (in %)	ANIb (aligned %)
		(%)	CI		
MTG105	*S. pseudopneumoniae* ATCC BAA-960^T^	56.0	53.3–58.7	0.48	93.69 (72.42)
	*S. pneumoniae* CCUG 28588^T^	52.1	49.5–54.8	0.70	92.92 (71.26)
	*S. toyakuensis* CCUG 75492^T^	51.3	48.6–53.9	0.24	92.79 (73.75)
	*S. mitis* NCTC 12261^T^	50.4	47.7–53.0	0.14	92.48 (74.90)
	"*S. symci*" C17^T^	48.6	46.0–51.2	0.41	91.75 (68.12)
	*S. oralis* ATCC 35037^T^	31.9	29.5–34.4	1.06	85.77 (64.32)

^*a*^ dDDH, digital DNA-DNA hybridization; ANIb, Average Nucleotide Identity based on BLAST; C.I, Confidence Interval.

### Biochemical feature and MALDI-TOF/mass analysis

Our findings suggested MTG105 to be a novel species, prompting a comparison of its biochemical features (Table 2). Alongside genomic analysis, the biochemical features of MTG105 were similar to those of *S. pneumoniae* and *S. pseudopneumoniae*. In clinical laboratory settings, optochin sensitivity and bile solubility testing are commonly used to identify *S. pneumoniae* (28, 30). Most *S. pneumoniae* exhibit susceptibility to optochin under both ambient air and 5% CO_2_ conditions. In contrast, *S. pseudopneumoniae* generally shows resistance under 5% CO_2_ conditions. Notably, MTG150 was susceptible under both conditions and was soluble in bile, similar to *S. pneumoniae*, which likely contributed to its initial identification as *S. pneumoniae*.

**TABLE 2:** Phenotypic characterization of strain MTG105 and related *Streptococcus* spp^*a*^

	MTG105	*S. pneumoniae* (SV 1)	*S. pseudopneumoniae* (O 108)	*S. mitis* (JCM 12971)
β-Glucosidase	−	v	−	−
β-Galactosidase	−	+	+	+
β-Glucuronidase	−	−	−	−
α-Galactosidase	−	+	−	+
Alkaline phosphatase	−	−	+	−
Alanyl-phenylalanyl-proline arylamidase	+	−	+	+
β-Galactosidase	−	v	−	−
Pyroglutamic acid arylamidase	−	+	−	−
*N*-Acetyl-β-glucosaminidase	−	+	−	−
Glycyl-tryptophan arylamidase	−	+	+	+
β-Mannosidase	−	−	−	−
Urease	−	−	−	−
Voges-Proskauer	−	−	−	−
Hydolysis of				
Hippurate	−	−	v	+
l-Arginine	−	v	−	−
Acid production				
d-Ribose	+	−	−	+
d-Mannitol	−	−	−	−
d-Sorbitol	−	−	−	−
d-Lactose	+	+	+	+
d-Trehalose	+	+	−	−
d-Raffinose	+	+	−	+
d-Sucrose	+	+	+	+
l-Arabinose	−	−	−	−
d-Arabitol	−	−	−	−
α-Cyclodexitrin	−	−	−	−
Glycogen	−	−	−	−
Pullulan	+	+	v	+
d-Maltose	+	+	v	+
d-Melibiose	−	−	−	−
d-Melezitose	−	−	−	−
Methyl-β-d-glucopyranoside	−	v	−	−
d-Tagatose	−	v	−	−

^*a*^ +, positive or grew; −, negative or not grew; v, variable; ND, no data.

In addition, MALDI-TOF/mass spectrometry analysis, which has been the standard identification method in clinical laboratories worldwide, showed that the best matches for this strain were *S. pneumoniae* DSM11866 DSM with a score of 2.000 and *S. pneumoniae* besST29 THL with a score of 1.970, difficult to distinguish from *S. pneumoniae*.

Considering both genomic and biochemical analyses, this isolate was proposed to be designated as *Streptococcus nakanoensis* sp. nov., representing a novel species within the genus *Streptococcus*.

### Pathogenicity of MTG105

**FIG 3:**
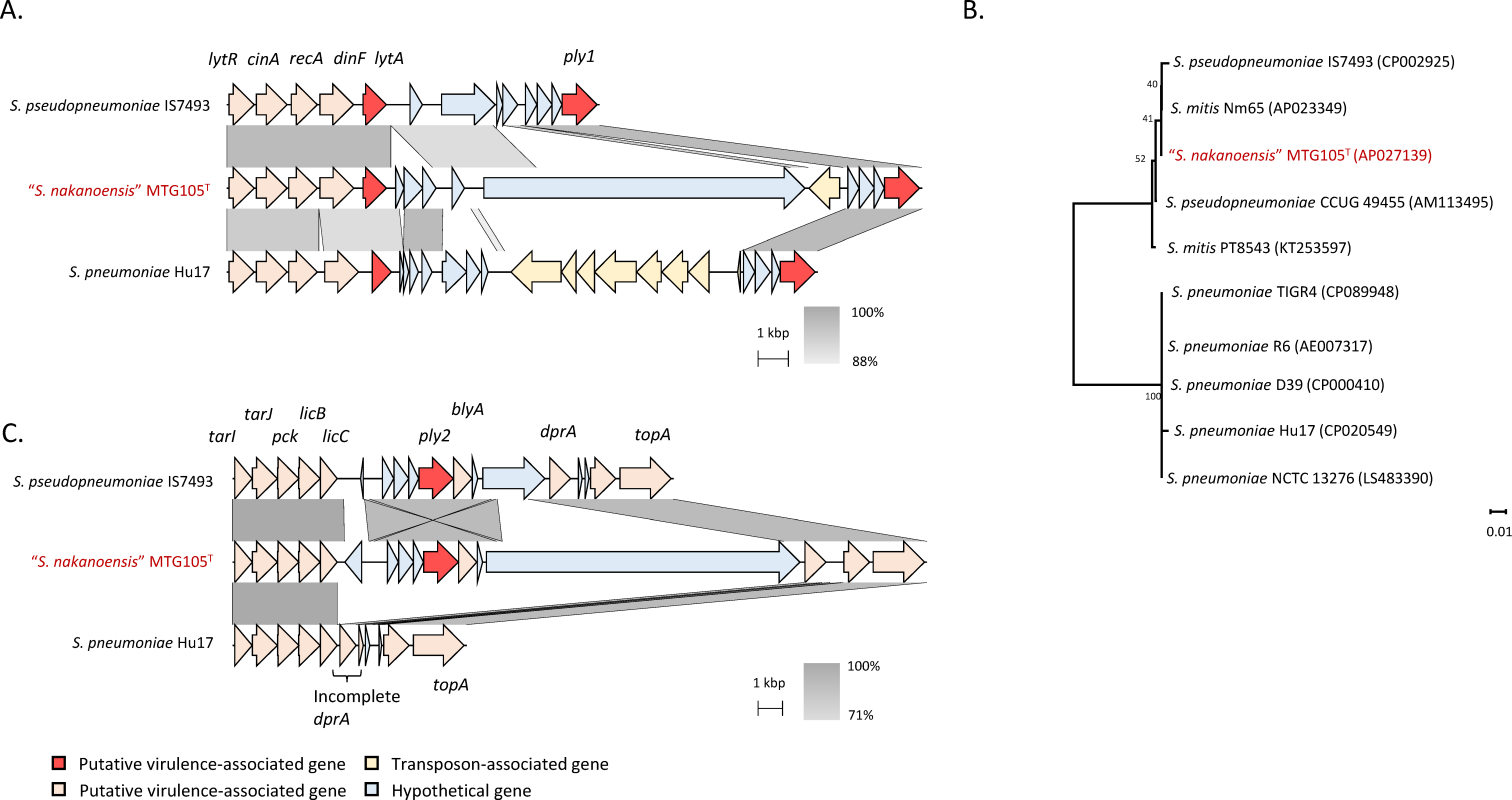
Virulence-associated genes of “*Streptococcus nakanoensis,*” *Streptococcus pneumoniae*, and *Streptococcus pseudopneumoniae*. (A and B) Comparative analysis of pneumolysin and its flanking regions. (A) *ply1* (*plyA*); (B) *ply2* (*plyB*). (C) Phylogenetic tree based on multiple alignments of *lytA* genes.

**FIG 4:**
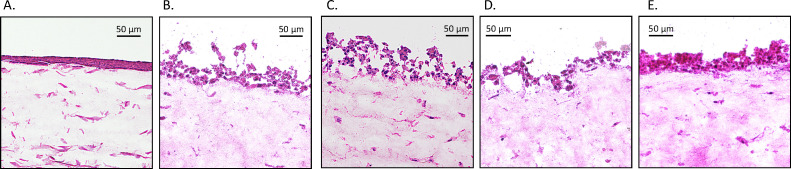
Pathogenicity of “*Streptococcus nakanoensis*”, *Streptococcus pneumoniae, Streptococcus pseudopneumoniae,* and *Streptococcus mitis* against 3D organotypic human lung tissue model. (A) No infection; (B) MTG105; (C) *S. pneumoniae* JPS 1346 (19F); (D) *S. pseudopneumoniae* GTC02864; and (E) *S. mitis* JCM 12971.

To characterize the virulence of MTG105, virulence-associated genes were explored using whole-genome data. Pneumolysin is recognized as a major virulence factor in both *S. pneumoniae* and *S. pseudopneumoniae* (31). Notably, MTG105 harbored two copies of the pneumolysin gene, similar to *S. pseudopneumoniae* (Fig. 3A). Phylogenetic analysis of pneumolysin-encoding genes revealed that pneumolysin in MTG105 clustered with that of *S. pseudopneumoniae* (Fig. 3B). To validate the pathogenicity of MTG105, a 3D human lung model system was infected with *S. nakanoensis* and related α-*Streptococcus* (Fig. 4) (24). Notably, *S. nakanoensis-*, *S. pneumoniae-,* and *S. pseudopneumoniae*-infected models showed a loss of dense layers and epithelial damage compared with the uninfected control and the *S. mitis*-infected model, suggesting that *S. nakanoensis* may have the same degree of pathogenicity as *S. pneumoniae* and *S. pseudopneumoniae*.

## DISCUSSION

Reports of novel species within α-*Streptococcus* have been increasingly documented. Although most of these species exist in the human oral cavity as commensal bacteria, they occasionally induce opportunistic infections in immunocompromised hosts (32–34). *S. pneumoniae* and *S. pseudopneumoniae* are well-characterized human pathogens. However, no other apparent pathogenic α-Streptococci have been identified. In this study, we isolated a quinolone-resistant α-*Streptococcus, S. nakanoensis* MTG105, from the sputum of a patient with pneumonia. Genetic analysis revealed that this species is closely related to *S. pseudopneumoniae*. In addition, the biochemical characteristics of MTG105 were similar to those of *S. pneumoniae* and *S. pseudopneumoniae*. Notably, this isolate exhibited susceptibility to optochin under both aerobic and 5% CO_2_ conditions, a characteristic that may lead to misidentification as *S. pneumoniae* in clinical laboratory settings.

The pathogenicity of this novel species remains unclear; consequently, future research will entail the analysis of its potential ability to induce infectious diseases in not only immunocompromised hosts but also healthy hosts. However, genome analyses revealed that MTG105 possessed two copies of pneumolysin, similar to *S. pseudopneumoniae*; the prevalence of the pneumolysin gene is a major virulence factor for both *S. pneumoniae* and *S. pseudopneumoniae* (35–37). In addition, infection assays using 3D human lung models demonstrated that MTG105 caused severe epithelial damage at the same level as *S. pneumoniae* and *S. pseudopneumoniae*, suggesting that this species should be regarded as pathogenic α-Streptococci, similar to *S. pneumoniae* and *S. pseudopneumoniae*.

Most α-Streptococci have a natural transformation ability, enabling them to take up extracellular DNA (38–40). This ability is also associated with the acquisition of antimicrobial resistance (41–44) and may contribute to the emergence of novel species. Although quinolone-resistant *S. pneumoniae* species are rare in clinical settings, the prevalence of quinolone-resistant isolates has seen a notable upsurge among other species occupying the same niche (21, 34, 45–48). In addition, quinolones in general are often used to treat elderly patients. This observation suggests that there may be barriers associated with developing quinolone resistance in *S. pneumoniae*. However, this novel species appeared to have overcome this barrier, suggesting that it might have evolved to adapt to its environment.

This species could potentially go unnoticed in clinical settings. In fact, if this isolate had not exhibited quinolone resistance, it would have been classified as *S. pneumoniae* without such analyses. Recently, a novel α-*Streptococcus*, *S. toyakuensis*, closely related to *S. mitis* and also exhibiting multidrug resistance, was identified (21). The report documented only one isolate; however, other recent studies have revealed that *S. mitis*/*oralis*-related species with reduced susceptibility to carbapenem encompassed *S. toyakuensis* (49), suggesting that *S. nakanoensis* in our study may also spread.

This study had several limitations. (i) We were unable to access detailed patient information and track outcomes. (ii) This species has been isolated only once to date. Therefore, its clinical significance remains unclear. Future genome epidemiological studies of α-*Streptococcus* will be necessary to identify the prevalence of this species. (iii) Although this species is classified as a novel species based on the criteria of dDDH and ANIb, it remains debatable whether these criteria are appropriate for application to a highly transferrable genus such as this.

Overall, in this study, a novel species classified into the *S. mitis* group was identified. This isolate may exhibit pathogenicity similar to that of *S. pneumoniae* and *S. pseudopneumoniae*. Furthermore, we propose that this species be designated as *S. nakanoensis* sp. nov.

### Description of *S. nakanoensis* sp. nov.

*S. nakanoensis* sp. nov. (derived from “*nakano,*” representing the city wherein the hospital of isolation is located and L. neut *ensis,* denoting place) is a gram-positive, facultative, catalase-negative, and non-spore-forming coccus. Colonies cultivated on blood agar containing horse or sheep blood were 0.25–0.5 mm in diameter and exhibited α-hemolysis after overnight culture at 37°C in ambient air. Growth was enhanced in the presence of 5% CO_2_. *S. nakanoensis* exhibited no Lancefield antigens. *S. nakanoensis* could use d-ribose, d-lactose, d-trehalose, d-raffinose, d-maltose, and pullulan and produce alanyl-phenylalanyl-proline arylamidase. *S. nakanoensis* exhibited susceptibility to optochin under both ambient air and 5% CO_2_ conditions. The optimum temperature for growth was 30–37°C.

The type strain, MTG105^T^ (= JCM 35953^T^ = CCUG 76894^T^), was isolated from human sputum.

## SUPPLEMENTAL MATERIAL

Figure S1 and Table S1 spectrum.01354-24-s0001.pdf Fig. S1: Multiple alignment of *gyrA* and *parC*. Table S1: Antimicrobial susceptibility of strain MTG105.

ASM does not own the copyrights to Supplemental Material that may be linked to, or accessed through, an article. The authors have granted ASM a non-exclusive, world-wide license to publish the Supplemental Material files. Please contact the corresponding author directly for reuse.
