# Supplementary material for: Molecular characterization of a novel putative pathogen, Streptococcus nakanoensis sp. nov., isolated from sputum culture
Source: Microbiol Spectr. 2024 Sep 13;12(10):e01354-24. doi: 10.1128/spectrum.01354-24 (PMC11465973; doi:10.1128/spectrum.01354-24)
Supplement: Figure S1 and Table S1 — Fig. S1: Multiple alignment of gyrA and parC. Table S1: Antimicrobial susceptibility of strain MTG105. [file spectrum.01354-24-s0001.pdf]

A. *gyrA*

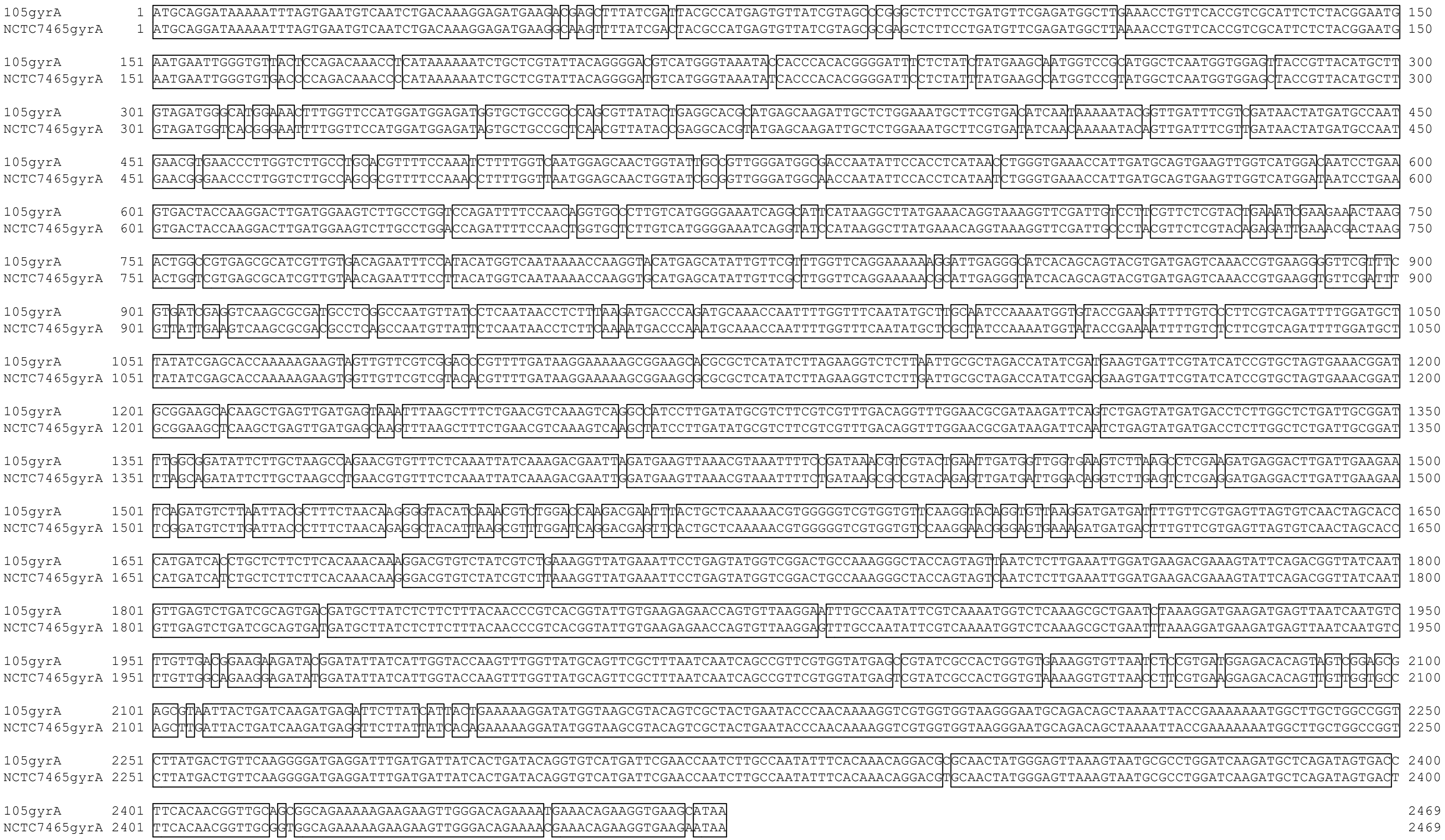

Supplement Figure 1. Multiple alignment of *gyrA* and *parC*

B. *parC*

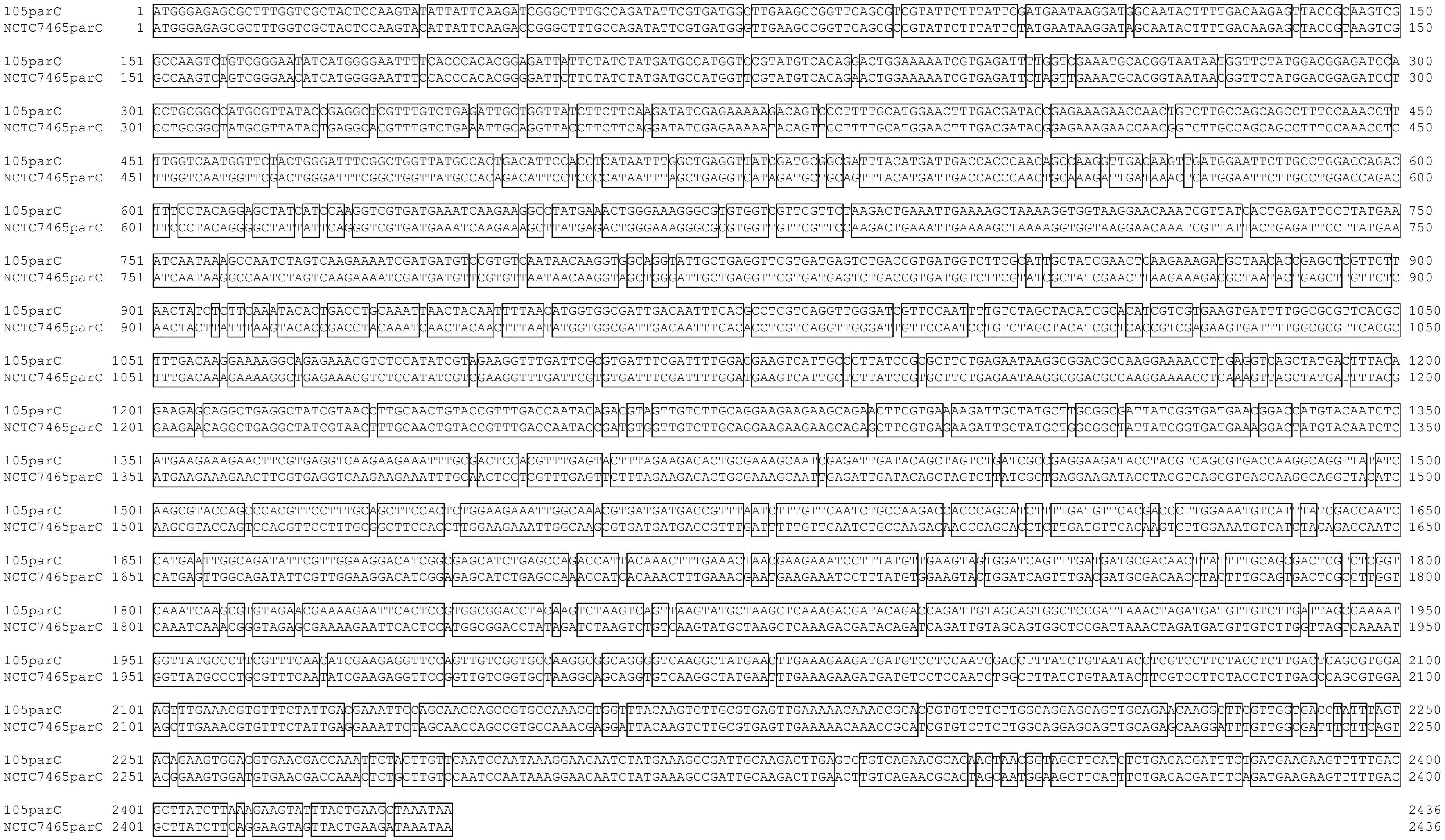

Supplement Figure 1. Multiple alignment of *gyrA* and *parC*

Supplement Table 1. Antimicrobial susceptibility of strain MTG105

Antimicrobial agent	MIC (µg/mL)	Decision*
Benzylpenicillin	< 0.063	Susceptible
Ampicillin	0.125	Susceptible
Cefotaxime	< 0.063	Susceptible
Meropenem	0.125	Susceptible
Tetracycline	32	Resistance
Levofloxacin	16	Resistance
Ciprofloxacin	32	Resistance
Clarithromycin	2	Resistance
Azithromycin	1	Intermediate
Vancomycin	0.5	Susceptible
Rifampicin	< 0.063	Susceptible

* Based on breakpoints of Streptococcus spp. viridance group from Clinical and Laboratory Standard Institute (CLSI) M100-S32
